# RETRACTION: Exosomal miR‐106a‐5p Accelerates the Progression of Nasopharyngeal Carcinoma Through FBXW7‐Mediated TRIM24 Degradation

**DOI:** 10.1111/cas.16312

**Published:** 2024-08-21

## Abstract

**RETRACTION**: C.‐W. Li, J. Zheng, G.‐Q. Deng, Y.‐G. Zhang, Y. Du and H.‐Y. Jiang, “Exosomal miR‐106a‐5p Accelerates the Progression of Nasopharyngeal Carcinoma Through FBXW7‐Mediated TRIM24 Degradation,” *Cancer Science* 113, no. 5 (2022): 1652–1668, https://doi.org/10.1111/cas.15337.

The above article, published online on 16 March 2022 in Wiley Online Library (wileyonlinelibrary.com), has been retracted by agreement between the authors; the journal Editor‐in‐Chief, Masanori Hatakeyama; the Japanese Cancer Association; and John Wiley & Sons Australia, Ltd. The retraction has been agreed following an investigation into concerns raised by a third party, which revealed that images of pathological results were duplicated across other articles in a different scientific context. The authors acknowledge the duplication of the images and apologise for the errors. Despite the authors providing all original data and reconducting the animal pathology experiments, given the extent and nature of duplications found, the editors consider the results and conclusion of this article to be invalid.

**RETRACTION**: C.‐W. Li, J. Zheng, G.‐Q. Deng, Y.‐G. Zhang, Y. Du and H.‐Y. Jiang, “Exosomal miR‐106a‐5p Accelerates the Progression of Nasopharyngeal Carcinoma Through FBXW7‐Mediated TRIM24 Degradation,” *Cancer Science* 113, no. 5 (2022): 1652–1668, https://doi.org/10.1111/cas.15337.

The above article, published online on 16 March 2022 in Wiley Online Library (wileyonlinelibrary.com), has been retracted by agreement between the authors; the journal Editor‐in‐Chief, Masanori Hatakeyama; the Japanese Cancer Association; and John Wiley & Sons Australia, Ltd. The retraction has been agreed following an investigation into concerns raised by a third party, which revealed that images of pathological results were duplicated across other articles in a different scientific context. The authors acknowledge the duplication of the images and apologise for the errors. Despite the authors providing all original data and reconducting the animal pathology experiments, given the extent and nature of duplications found, the editors consider the results and conclusion of this article to be invalid.

